# An Interdisciplinary Approach to Quantify the Human Disaster Risk Perception and Its Influence on the Population at Risk: A Case Study of Longchi Town, China

**DOI:** 10.3390/ijerph192416393

**Published:** 2022-12-07

**Authors:** Shengnan Wu, Yu Lei, Wen Jin

**Affiliations:** 1 Chongqing Economic and Social Development Research Institute, Chongqing 400041, China; 2 Key Laboratory of Mountain Hazards and Earth Surface Processes, Institute of Mountain Hazards and Environment, Chinese Academy of Sciences, Chengdu 610041, China; 3 China-Pakistan Joint Research Center on Earth Sciences, Chinese Academy of Sciences-Higher Education Commission (CAS-HEC), Islamabad 45320, Pakistan; 4 University of Chinese Academy of Sciences, Beijing 100049, China; 5 National Disaster Reduction Center of China, Ministry of Emergency Management, Beijing 100084, China

**Keywords:** disaster risk reduction, disaster risk perception, the population at risk, agent-based modeling

## Abstract

Understanding disaster risk perception is vital for community-based disaster risk reduction (DRR). This study was set to investigate the correlations between disaster risk perception and the population at risk. To address this research question, the current study conducted an interdisciplinary approach: a household survey for measuring variables and constructed an Agent-based model for simulating the population at risk. Therefore, two correlations were defined, (1) between risk perception and willingness to evacuate, and (2) between willingness to evacuate and the population at risk. The willingness to evacuate was adopted as a mediator to determine the relationship between risk perception and the population at risk. The results show that the residents generally have a higher risk perception and willingness to evacuate because the study area frequently suffered from debris flow and flash floods. A positive correlation was found between risk perception and willingness to evacuate, and a negative correlation to the population at risk. However, a marginal effect was observed when raising public risk perception to reduce the number of the population at risk. This study provides an interdisciplinary approach to measuring disaster risk perception at the community level and helps policymakers select the most effective ways to reduce the population at risk.

## 1. Introduction

### 1.1. Background

Earthquakes, landslides, debris flows, and other types of disasters have posed significant threats to human safety, property, and critical infrastructures [1,2,3]. The Centre for Research on the Epidemiology of Disasters (CRED) reported that just in 2021, there were 432 disaster events around the world, accounting for 10,492 deaths, affecting 101.8 million people [4].

Global landmark agendas, including the Sendai Framework for Disaster Risk Reduction 2015–2030 (SFDRR), the 2030 Agenda for Sustainable Development (SDGs), and the Paris Agreement, highlight the importance of adopting disaster risk reduction (DRR) measures to help reduce the loss of life and property damage. With the deepening understanding of disaster risk, people’s response strategy to disasters has undergone a conceptual change, and the focus of the strategy has gradually shifted from the traditional passive response to disasters to taking action for DRR actively. In this process, it has also been discovered that in addition to developing technology and techniques [5,6,7,8,9], it is important to consider human dynamic factors in DRR [10], such as risk perception, willingness to evacuate, and emergency behaviors [11,12,13].

Public risk-reducing behaviors and risk perception are interlinked to each other. People with higher risk perception are more likely to take actions to reduce or avoid disaster risks [14,15]. As one of the key factors that may influence public risk-reducing behaviors [16], risk perception has become an important research agenda in DRR and plays a pivotal role in ensuring the effectiveness of DRR measures [17,18,19]. However, to date, there is no unified standard for measuring disaster risk perception in the academic community. Many factors may affect public risk perception, and various methods have been developed and introduced to measure risk perception, such as disaster characteristics [11], distance to disasters [20], demographic factors [11,20,21], gender differences [22], and disaster experience [20]. Meanwhile, although mainstream schools believe that higher risk perception can encourage people to take DRR actions [23,24], it is difficult to quantify the impacts of risk perception on DRR. Therefore, the primary aim of this paper is to explore the correlation between risk perception and the population at risk, which may provide empirical evidence for how to reduce disaster risks by affecting public risk perception.

### 1.2. Research Question and Hypothesis

**Figure 1:**
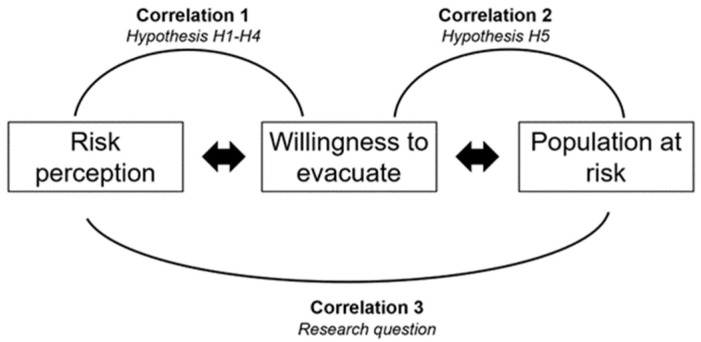
Research question and hypothesis.

This study primarily set the research question as the correlation between public risk perception and the population at risk (Figure 1). Understanding the complexity of risk perception is vital, and different authors have measured risk perception in various ways. As the current study chose a Chinese town as a study site, we sorted out the existing research in risk perception measurement that set China as a case. We found that some scholars divide risk perception into disaster intensity, disaster loss, environmental sensitivity, and the characteristics of the affected population [25]. Some scholars also believe that disaster risk perception includes the likelihood, threat, knowledge, and attitude of resistance to disasters [13] or disaster characteristics, disaster experience, disaster knowledge, and risk communication [26]. Therefore, for reliability and validity of the questionnaire, exploring the existing studies in the same study area [13,26,27,28] and considering the actual situation in the study area, we classified disaster risk perception into four variables, namely (a) knowledge of disasters, (b) impacts of disasters, (c) participation in DRR, and (d) disaster experience, and measured the comprehensive risk perception of disasters by summing up four variables. Subsequently, this study sorted out the public willingness to evacuate and defined the possible correlations between the public willingness to evacuate and the four risk perception variables. Therefore, the research hypotheses *H1*–*H4* were proposed.

(a) Knowledge of disasters: Knowledge of disasters refers to residents’ knowledge of disasters, including the main types of local hazards, escape routes, and access to warning information. Many pieces of the literature confirmed the significance of knowledge for public risk perception [20,29]. The more the residents know about the major local hazards, the higher the risk perception they may have, and thus the more likely they are to take proactive measures to cope with the hazards [30]. Evacuation before disasters is one of the active measures to cope with disasters. Therefore, hypothesis *H1* is proposed:

*H1: Knowledge of disasters had a significant positive effect on willingness to evacuate*.

(b) Impacts of disasters: Impacts of disasters refer to how the residents perceive the level of disaster threat to their personal and local lives, property, production, and livelihood. Most studies have concluded that residents with stronger perceptions of disasters impacts of disasters tend to have a stronger willingness to evacuate [31,32,33]. For instance, people who live closer and may have a larger impact on the disaster may have higher risk perception [20,29,34]. However, some studies have also shown that disasters’ impact levels and risk perception are not necessarily significantly related [35]. Based on this analysis, research hypothesis *H2* is proposed.

*H2: Impacts of disasters had a significant positive effect on the willingness to evacuate*.

(c) Participation in DRR: Participation in DRR refers to the willingness and cooperation of residents to participate in local disaster risk reduction practices. Literature reveals that government officials and residents vary greatly in risk perception [20]. Residents actively participating in local DRR activities tend to have higher risk perceptions [36]. Therefore, participation in DRR can be measured indirectly by measuring residents’ participation in DRR and, thus, risk perception. Based on this analysis, research hypothesis *H3* is proposed.

*H3: Participation in DRR had a significant positive effect on the willingness to evacuate*.

(d) Disaster experience: Disaster experience refers to the number of disasters an individual has experienced. Disaster experience is one of the important factors affecting risk perception [20,26]. Many works of literature reveal that residents who have experienced disasters multiple times or have experienced large disasters tend to have higher perception levels [20,34,37]. Based on this analysis, research hypothesis *H4* was proposed.

*H4: Disaster experience had a significant positive effect on willingness to evacuate*.

Subsequently, this study developed an agent-based model to simulate the population at risk under different willingness to evacuate. It investigated the correlation between the population at risk and willingness to evacuate. The higher willingness to evacuate provides more time for evacuation and thus is more likely to reduce the overall number of local casualties due to disasters [13,33]. On the contrary, residents are less willing to evacuate and are unwilling to take the initiative to evacuate to a safe area immediately after receiving warning information. In this case, they can only passively escape from dangers when they encounter a disaster, which tends to have a lower survival rate. The population at risk refers to those residents who may be faced with dangers in disaster events. Therefore, hypothesis *H5* is proposed:

*H5: Public risk perception had a significant positive effect on reducing the population at risk*.

Once these two correlations, (1) between the willingness to evacuate and the risk perception and (2) between the public willingness to evacuate and the population at risk, were extracted, we can adopt the willingness to evacuate as a mediator to link risk perception and the population at risk. This study, therefore, explored the correlation between risk perception and the population at risk.

## 2. Materials and Methods

### 2.1. Data Preparation

#### 2.1.1. Study Area

**Figure 2:**
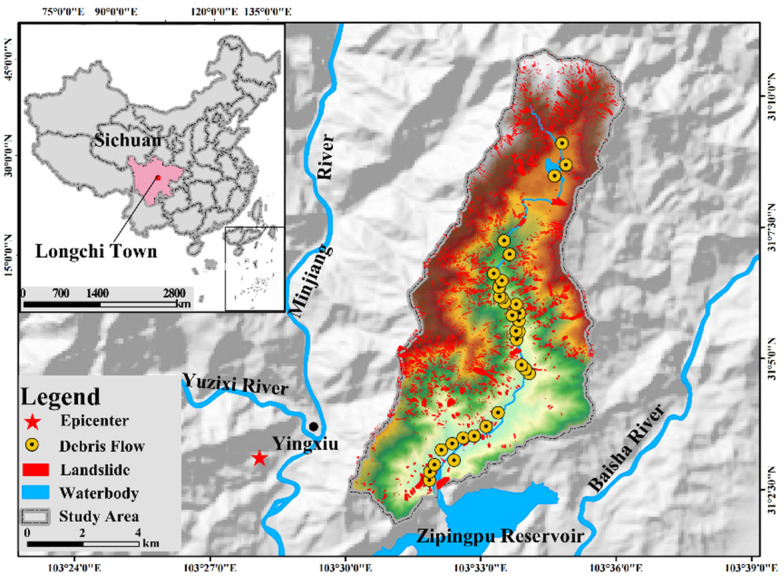
Location of Longchi town.

This study selected Longchi township as the study site (Figure 2). Longchi township is in Dujiangyan City, Sichuan Province, China, which is 80 km away from Chengdu. It is the largest township in Dujiangyan, with five communities, including Chaguan, Yunhua, Liping, Nanyue, and Dongyue. Many natural and tourism resources, such as Dujiangyan National Forest Park, Giant Panda World Heritage Site, and Longxi-Hongkou National Nature Reserve, are located within Longchi town. According to statistics, before the 2018 Wenchuan earthquake, the output value of the primary and tertiary industries reached over 3,487,500 USD, and the fiscal revenue exceeded 159,030 USD.

Unfortunately, the 2018 Wenchuan earthquake struck the town and locked down its industries. During the earthquake, Longchi was devastatingly hit, with 36 people dead, 17 missing, and 1558 injured due to it occurring only three kilometers from the epicenter, Yingxiu town. Meanwhile, its roads, electricity, and communications were shut down, and the tourist resort suddenly became isolated. After the earthquake, due to the post-earthquake fractured rock and loose soil on the steep slopes [38], there were a total of 217 seismic hazard sites, 137 threatening farming sites, 77 threatening road safety sites, and 4239 threatening people [26]. These disasters after the earthquake repeatedly hit its pillar industry, tourism, as the National Forest Park was closed to the public.

Among the secondary disaster events after the earthquake, the debris flow and flash flood on 13 August 2010 (the “813” disaster event) was one of the most destructive and typical disasters. More than 50 gullies in the area were affected by flash floods and debris flows, which destroyed many houses and roads and caused 495 casualties and huge economic losses. Thus, the current study takes the “813” disaster event as a case to simulate the population at risk and test the hypothesis.

#### 2.1.2. Survey Design

This study used questionnaires and semi-structured interviews to collect data. Questions were asked about the four variables of risk perception (knowledge of disasters, impacts of disasters, participation in DRR, disaster experience) and willingness to evacuate, considering the study area’s actual situation and the study participants. Additionally, respondents who had experienced the “813” disaster event were asked about their actual emergency behaviors at that time. In addition, the questionnaire included personal information about the residents, such as gender, age, and occupation.

In this study, we first conducted a focus group interview with 13 residents to gain an in-depth understanding of their risk perception variables, willingness to evacuate, and behaviors on the “813” disasters to adjust the survey questions and layout. The follow-up survey was conducted in May 2020 with a total of 166 respondents in the Longchi township. Each question survey lasted for half an hour. The targeted samples were taken separately from the whole area of Longchi township, which covered nearly 50% of resident households, and were widely distributed in locations, professions, gender, and age.

The basic information of the sample is shown in Table 1, in which the proportion of male and female residents in the sample was equal, with slightly more males than females. The age of the sample was predominantly middle-aged and elderly (59.26%), between 41 and 60 years old. They were mainly engaged in self-employment (37%) and agriculture and forestry (38.9%). The sample structure is consistent with the basic situation of Longchi town and is representative.

### 2.2. Measuring Variables

#### 2.2.1. Risk Perception

As discussed in Section 1.2, Research Question and Hypothesis, for reliability and validity of the questionnaire, exploring the existing studies in the same study area [13,26,27,28] and considering the actual situation in the study area, we classified disaster risk perception into four variables, namely (a) knowledge of disasters, (b) impacts of disasters, (c) participation in DRR, and (d) disaster experience. The sum of the four variables was calculated to measure the public disaster risk perception, and each variable was measured separately (Table 2). Meanwhile, the value of Cronbach’s alpha for each variable is 0.6 on average, which indicates a good agreement between entries.

#### 2.2.2. Willingness to Evacuate

For measuring the public willingness to evacuate in the face of a disaster, a short questionnaire was designed to ask participants to rate how strongly they agreed with the statement: Would you be willing to evacuate when a disaster threatens your place? The answers to the questionnaire were measured using a 5-point Likert scale: 1 = strongly disagree, 2 = disagree, 3 = neutral, 4 = agree, 5 = strongly agree.

### 2.3. Simulating Population at Risk

#### 2.3.1. Agent-Based Modeling Design

Agent-based modeling (ABM) is a microscopic to macroscopic modeling approach based on the systems theory. With a multidisciplinary intersection background, it is widely used in mathematics, physics, biology, sociology, and other fields [39]. It has also been introduced to disaster risk assessment recently [10,40,41], especially for behavioral decisions on disaster response that fully consider the diversity of the population.

**Figure 3:**
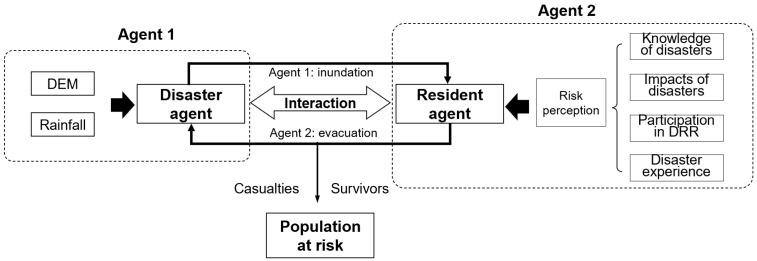
Interactions of agents in the ABM model.

The current study takes advantage of the ABM modeling approach and relies on the Netlogo platform language programming to develop a model for simulating at-risk populations. The model inputs the real geospatial environment and generates many different types of agents (resident agents, disaster agents, building agents, etc.) (Figure 3).

Resident agents: This type of agent is set to simulate different risk-reducing behaviors of each resident. Each agent can display autonomously in the system and has its own physical attributes (age, gender, vision, stamina, safety) and mental attributes (four variables of risk perception). Data are obtained from the Sixth National Population Census of the People’s Republic of China [42] and the survey investigation.

Disaster agents: This agent refers to the flash floods and debris flows in the “813” disaster event and can simulate the inundation areas dynamically. Their attributes include locations and debris flow volumes.

All agents are distributed in a 2D space according to established rules. They can act autonomously according to the set rules, perform activities, and interact with other agents and the surrounding environment (patches). The behaviors of all subjects occur in parallel. They are updated asynchronously, through which it is possible to simulate the behaviors of various types of residents and other agents in the event of a disaster. The model can measure the population at risk under different scenarios of willingness to evacuate by reproducing the entire emergency response process from the bottom up.

#### 2.3.2. Experiment Process

**Figure 4:**
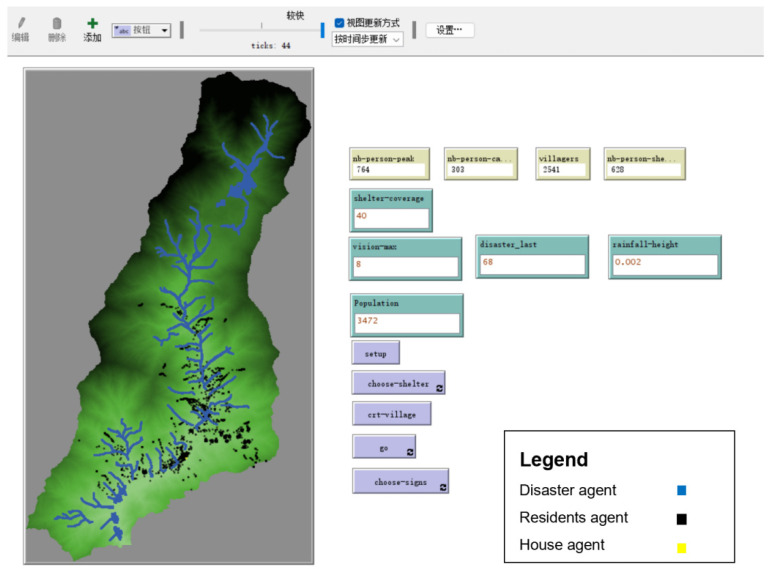
Screenshot of simulation on Netlogo platform.

The Netlogo platform (Figure 4) was used to reproduce the “813” disaster event in Longchi town according to the natural and socioeconomic conditions and to visually assess the population at risk under different willingness to evacuate scenarios. For each willingness scenario, 50 independent repeated experiments were conducted. Before starting the scenario simulation, the model was validated by using a questionnaire to obtain the willingness to evacuate through a survey of respondents who experienced the “813” disaster event and inputting the data into the simulation platform to verify the accuracy of the model. The repeated simulation results were projected to be 481 casualties on average, which was consistent with the real situation (486 casualties) and indicated that the risk assessment model has good reliability.

## 3. Results

### 3.1. Descriptive Statistics

#### 3.1.1. Risk Perception

**Figure 5:**
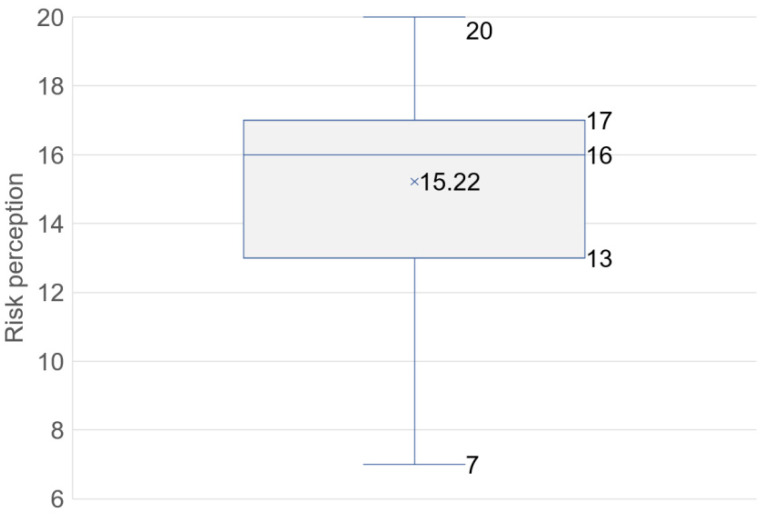
Measurement of risk perception.

**Figure 6:**
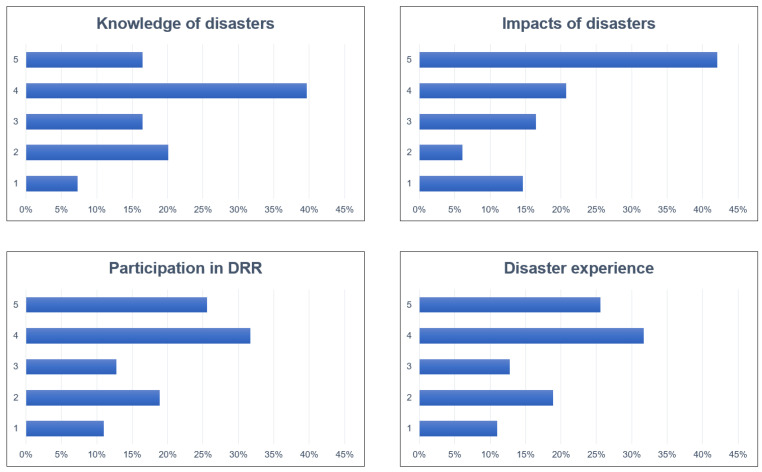
Statistic distribution of each variable of risk perception (1 = strongly disagree, 2 = disagree, 3 = neutral, 4 = agree, 5 = strongly agree).

As mentioned in Section 2, Materials and Methods, the four variables of public risk perception, namely, (a) knowledge of disasters, (b) impacts of disasters, (c) participation in DRR, and (d) disaster experience, were added to calculate the overall risk perception. The results are shown in Figure 5. The risk perception values were in the range of 0–20. The value of Cronbach’s alpha for each variable is 0.6 on average, which indicates a good agreement between entries. The overall level of public risk perception is moderately high, and 50% of the respondents perceived the risk as between 13 and 17. Specifically, the maximum value is 20, the minimum value is 7, the mean value is 15, and the variance is 8.01. The results of each variable are displayed in Figure 6

Knowledge of disasters: The survey results show that overall public knowledge of disasters is high. Among them, 56% of the respondents are relatively or very well informed. However, 27% of the respondents still have insufficient knowledge of disasters, especially about how to escape from disasters.

Impacts of disasters: Respondents believe disasters will have a greater impact on their lives and property, as 63% of the respondents thought the level of impact was very high or relatively high. In contrast, only 21% thought the impact level was very low or relatively low, and 16% remained neutral. Meanwhile, 76% of respondents believe disasters impact the township more than individuals.

DRR participation: The survey results show that residents’ participation in DRR is high, with 58% of respondents having relatively high or very high participation in DRR and 30% having low or very low participation in DRR.

Disaster experience: The survey results show that most residents have experienced multiple large-scale disasters. Among them, 98% of the respondents have experienced the Wenchuan earthquake and secondary disasters, and only three have ever experienced disasters. This result is consistent with how the Wenchuan earthquake affected the local area.

#### 3.1.2. Willingness to Evacuate

**Figure 7:**
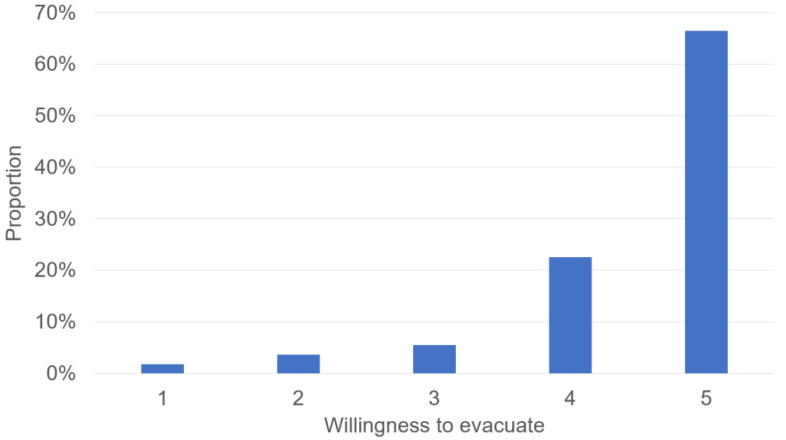
Statistic distribution of willingness to evacuate (Question: Would you be willing to evacuate when a disaster threatens your place? Answer: 1 = strongly disagree, 2 = disagree, 3 = neutral, 4 = agree, 5 = strongly agree).

From the frequency distribution of public willingness to evacuate (Figure 7), it can be seen that Longchi town residents are strongly willing to evacuate when disaster strikes. Specifically, 66% of the 164 respondents said they were very willing to evacuate in the event of a disaster, and 23% were willing to evacuate. In contrast, only 6% of residents said they were reluctant to evacuate, and 5% of residents were neutral about whether to evacuate immediately in the event of a disaster.

### 3.2. Defining the Correlations

#### 3.2.1. Between Risk Perception and Willingness to Evacuate

In this study, regression analysis was used to reveal the correlation between risk perception (including four variables respectively) and willingness to evacuate by using SPSS26, by which to verify the research hypothesis *H1*–*H4* mentioned in Section 1.2. The ANOVA results showed that the overall significance test statistic was at the 1% level, which indicated that a follow-up analysis could be conducted.

**Figure 8:**
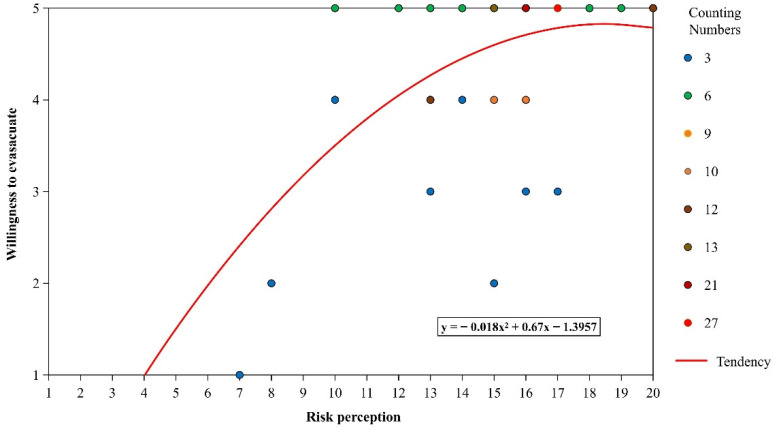
Correlation between risk perception and willingness to evacuate.

The regression analysis results of risk perception and willingness to evacuate are shown in Table 3. Meanwhile, their correlation is displayed in Figure 8. Resident risk perception was positively and significantly correlated with their willingness to evacuate (f = 52.542, *p* < 0.001). Therefore, the higher the disaster risk perception, the stronger their willingness to evacuate. More specifically, for each 1-unit increase in risk perception, the willingness to evacuate increases by an average of 0.157 units. Among the four variables of risk perception, three variables were all positively and significantly correlated with willingness to evacuate, namely (a) knowledge of disasters, (b) impacts from disasters, and (c) participation in DRR. Specifically, for each unit of increase in knowledge of disasters and participation in DRR, the public willingness to evacuate increased by 0.193 and 0.275 units, respectively. For each unit increase in impacts of disasters, there is a corresponding increase of 0.086 units in their willingness to evacuate. Therefore, the higher the value of these three variables the residents may have, the more willing they are to evacuate. Meanwhile, there was one surprising variable of disaster risk perception, namely (d) disaster experience, which was not significantly related to willingness to evacuate.

#### 3.2.2. Between Willingness to Evacuate and the Population at Risk

**Figure 9:**
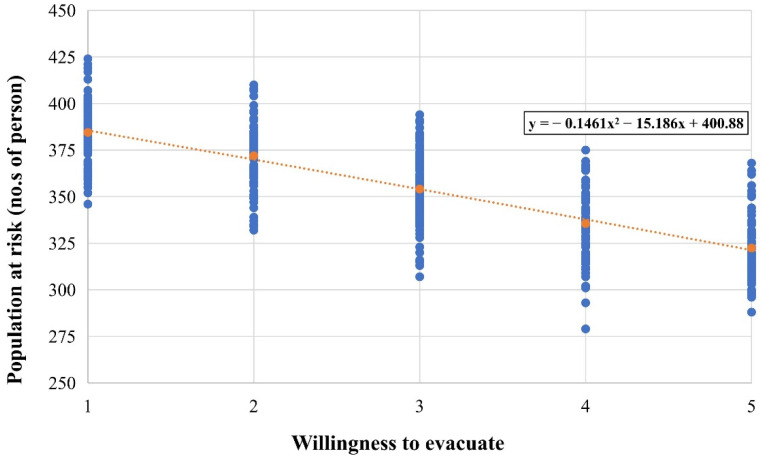
Correlation between willingness to evacuate and the population at risk.

The ABM model projected the correlation between willingness to evacuate and the population at risk (Section 2.3). Figure 9 presents the population at risk under different scenarios of willingness to evacuate, which can be indicated that with successive increases in the willingness to evacuate, the population at risk gradually decreased. Specifically, the average number of casualties decreased from 384.48 (willingness = 1) to 354.11 (willingness = 3) and 322.41 (willingness = 5). The number of casualties decreased by 8% versus 16%.

#### 3.2.3. Between Risk Perception and the Population at Risk

**Figure 10:**
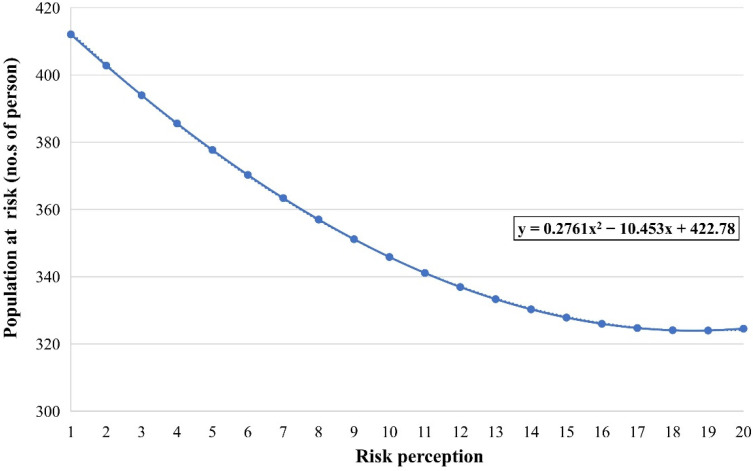
Correlation between risk perception and the population at risk.

Willingness to evacuate playing as a mediator, the correlation between risk perception and the population at risk is investigated from two correlations obtained previously in Section 3.2.1 and Section 3.2.2. Figure 10 provides the scatter diagram of the relationship between risk perception and the population at risk. It shows that there has been a gradual decline in populations at risk at the rate of 21.2%. Specifically, the number of populations at risk has been reduced from 412 to 325, with a gradual increase in risk perception.

In addition, although there is an overall trend of decline in the number of populations at risk, the rate of decline is different in three stages. When the risk perception increased from 1 to 6, the decline rate was over 2%. Subsequently, the rate drops to around 1% and less than 1% as the risk perception reaches 13.

## 4. Discussion

The results of the study show that most residents were willing to evacuate before disasters (Figure 7). One possible reason for the high level of willingness is owed to the government’s efforts over the years after the Wenchuan earthquake in 2008. The local government has recently attached great importance to DRR [26]. After the Wenchuan earthquake, Longchi town gradually established a good mechanism for dealing with local geohazards. Longchi town organizes various DRR activities every year, for example, holding a town-wide flood prevention and geohazard mobilization meeting at the beginning of the year and conducting flash flood and geohazard warning training and emergency drills. These activities allow all stakeholders to actively participate in the local disaster prevention and mitigation efforts, including officials, local DRR practitioners, hospitals, schools, and individuals. In other words, this may be evidence that the efforts of community governments are useful in raising the public willingness to willingness and may provide practitioners with more confidence and motivation to accomplish community DRR.

Subsequently, this study was set to define three correlations. The correlation between risk perception and willingness to evacuate was set to demonstrate the *H1*–*H4* (Section 1.2). Among them, the findings are consistent with the research hypotheses *H1* and *H3*, where (a) knowledge of disasters and (c) participation in DRR strongly correlate with their willingness to evacuate (Figure 8). The possible reason for this is that after the Wenchuan earthquake, residents were regularly trained in disaster prevention and mitigation knowledge and skills and were motivated to participate in local disaster prevention and mitigation efforts through incentives. These DRR measurements brought people knowledge and awareness about disasters, consequently making them more willing to evacuate when faced with disasters. Several studies also indicated that knowledge [29,30,36] and participation in community DRR [20,36] have positive impacts on public risk perception.

Meanwhile, though it is found that (b) impacts of disasters are consistent with the research hypotheses H2, their correlation seems slightly less (Figure 8). This result is slightly different from the previous studies as most believed the impacts of disasters, such as distance to disasters, had strong significance to risk perception and associated actions [20,29,43]. One possible reason is related to the fact that most respondents may believe that the degree of impact of disasters on the Longchi township is higher than that of themselves. Thus, although residents are aware of the dangers of disasters, the correlation of this dimension with a willingness to evacuate is relatively lower than that of other dimensions because they do not perceive the impacts of disasters to be higher for themselves.

However, only one variable of disaster risk perception, namely (d) disaster experience, was not significantly related to willingness to evacuate. This result is inconsistent with research hypothesis *H4*. Although much previous literature [20,27,34,37] reveals that public past disaster experience is positively associated with risk perception and related actions, our study obtains an opposite finding in this variable. The possible reason is that this study was conducted with the residents of Longchi town as the study population. Longchi is adjacent to the epicenter of the Wenchuan earthquake, and most respondents have experienced the Wenchuan earthquake and the series of secondary disasters after the earthquake. For almost all interviewees, similar disaster experiences left a consistent and deep psychological feeling. Additionally, the study was designed to investigate the correlation between willingness to evacuate and the at-risk population. The result in Figure 9 was deployed by the ABM model and showed that the increasing willingness to evacuate could significantly reduce the population at risk, supporting hypothesis 5.

For the research question, this study aimed to understand the correlation between risk perception and the at-risk population. The result in Figure 10 shows an overall trend of decline in the number of populations at risk when the public risk perception increases, which provides scientific evidence for policymakers that the public disaster risk perception should be raised. Improving disaster risk perception can enable residents to be more willing to evacuate, thus effectively reducing casualties during the disaster. Meanwhile, raising public disaster risk perception is relatively low-cost compared with engineering measurement [36]. For example, low-cost engineering measures, such as disaster education and community DRR activities, can effectively improve public risk perception. Therefore, this approach to improving risk perception can be replicated in those communities in developing countries threatened by disasters. In terms of specific DRR measures, the government can carry out a variety of education and training, including the knowledge on the prevention and treatment of main disaster types and capacities to avoid and save themselves. In addition, a multi-stakeholder DRR participation system can be established to increase the risk perception of all relevant stakeholders, such as schools, hospitals, and NGOs [44]. Residents can improve their risk perception and reduce population risk by participating in community DRR activities.

Meanwhile, one interesting finding is that there is a marginal effect of raising public risk perception on reducing population risk. This phenomenon may be because when most of the residents have a high level of risk perception, other factors can also influence the risk of the population, such as the time of early warning, evacuation patterns, and different vulnerabilities. Since there is a marginal effect for raising risk perception in DRR when most residents have a high level of risk perception, the government can dedicate a portion of the investment to other aspects that are likely to affect public emergency behavior. For example, there are differences in vulnerability between populations. Disadvantaged populations, such as the disabled, pregnant women, and the elderly, are more vulnerable during disasters [12,45]. They usually need to take a longer time for evacuation or even need help from the government to evacuate to safe places in a timely manner. In this case, the government may use partial investment to tailor DRR measures for disadvantaged populations after most people have a higher risk perception.

## 5. Conclusions

This study has tried to combine interdisciplinary methods from both social sciences and natural sciences, including survey questions, statistical analysis, and ABM models, to establish a correlation between disaster risk perception and the population at risk. This attempt may provide some methodologies and ideas for subsequent interdisciplinary studies. In this paper, the following conclusions were obtained by measuring risk perception, willingness to evacuate, and the population at risk and exploring the correlation between the above.

Residents generally have a higher risk perception and willingness to evacuate. The results showed that 50% of the respondents had risk perception levels between 13 and 17 (maximum 20), and 66% of the residents indicated they were very willing to evacuate in the event of a disaster. The increased risk perception and willingness are inseparable from local investments in disaster education and other efforts in recent years. This result, to some extent, reflects the effectiveness of local government investment in DRR in recent years.

A positive correlation was found between risk perception and willingness to evacuate. For every 1 unit increase in risk perception, willingness to evacuate increased by an average of 0.157 units. Regarding each risk perception variable, three variables, namely (a) disaster risk perception, (b) knowledge of disasters, and (c) participation in DRR, have a significant positive correlation with the willingness to evacuate, supporting hypotheses *H1*, *H2*, and *H3*. However, the results between (d) disaster experience and the willingness to evacuate were not statistically significant, which does not correspond to hypothesis *H4*.

The ABM simulation results indicated that as the willingness to evacuate rises, the population at risk decreases rapidly (up to 16%). The correlation between willingness to evacuate and the population at risk can support hypothesis H5.

The correlation between risk perception and the population at risk also provided a positive significance. The observations have indicated a serious decline in the population at risk from 412 to 325 when their risk perception rises from 1 to 20. However, the rate of decline becomes slower after their risk perception reaches 13, which indicates a marginal effect of raising public risk perception on reducing the risk of population

These findings have significant implications for understanding public risk perception and how to reduce disaster risks by changing public risk perception. Firstly, the public risk perception should be increased since research findings show that increased public risk perception is associated with a higher willingness to evacuate, which can effectively reduce the population at risk. Moreover, raising risk perceptions is often low-cost and affordable for most communities. Therefore, it is recommended that most communities can conduct education to increase public risk perception and thus reduce the number of casualties at the community level to some extent. Secondly, although it is advocated from all levels to strengthen community disaster mitigation, it is nevertheless not requiring communities to invest heavily all at once. A large investment without a goal may not necessarily achieve very good results. Therefore, at the community level, what needs to be carried out is policy optimization, using the results of scientific research to provide more effective disaster reduction measures based on a combination of local natural, social, and economic conditions.

## Figures and Tables

**Table 1:** Sample distribution.

Items	Categories	Proportion (%)
Gender	Male	60
	Female	41
Age	18–25	2
	26–30	7
	31–40	6
	41–50	26
	51–60	33
	Over 60	26
Occupation	Business	37
	Farming	39
	Others	24

**Table 2:** Index for measuring public risk perception.

Variables		No.	Variable Description and Definition
a	knowledge to disasters	A1	I know the main types of disasters in my community.
		A2	I know how to escape from these disasters.
		A3	I have access to disaster information, including early warnings, warning signs, evacuation routes.
b	Impacts of disasters	B1	Disasters can harm me.
		B2	Disasters can have a serious impact on my properties.
		B3	Disasters can have a serious impact on Longchi town.
c	Participation in DRR	C1	I would like to participate in local DRR activities.
		C2	I have participated in many DRR activities.
		C3	If I receive an early warning, I am willing to cooperate with the community for DRR.
d	Disaster experience	D1	I have experienced disasters many times.

Note: The answers to the questionnaire were measured using a 5-point Likert scale: 1 = strongly disagree, 2 = disagree, 3 = neutral, 4 = agree, 5 = strongly agree.

**Table 3:** Regression analysis between risk perception and willingness to evacuate.

Variables	Willingness to Evacuation				
	β	Standard Error	F	R²	Adjusted R²
risk perception	0.157 ***	0.022	52.542	0.245	0.240
knowledge to disasters	0.193 ***	0.051	22.478	0.361	0.345
Impacts of disasters	0.086 **	0.042			
Participation in DRR	0.275 ***	0.046			
Disaster experience	−0.142	0.080			

Note: *** means significant at less than 1% probability ** means significant at less than 5% probability.

## Data Availability

The data presented in this study are available on request from the corresponding author. The data are not publicly available due to funding project policy.
